# Why it is crucial to analyze non clonal chromosome aberrations or NCCAs?

**DOI:** 10.1186/s13039-016-0223-2

**Published:** 2016-02-13

**Authors:** Henry H. Q. Heng, Sarah M. Regan, Guo Liu, Christine J. Ye

**Affiliations:** Center for Molecular Medicine and Genetics, Wayne State University School of Medicine, Detroit, MI 48201 USA; Department of Pathology, Wayne State University School of Medicine, 3226 Scott Hall, 540 E. Canfield, Detroit, MI 48201 USA; Division of Graduate Medical Sciences, Boston University School of Medicine, Boston, MA 02118 USA; The Division of Hematology/Oncology, University of Michigan Comprehensive Cancer Center, Ann Arbor, MI USA

**Keywords:** Clonal Chromosome Aberrations or CCAs, Fuzzy inheritance, Genome instability, Genome theory, Heterogeneity, Non Clonal Chromosome Aberrations or NCCAs, NCCA/CCA cycle, Parts inheritance, System inheritance

## Abstract

Current cytogenetics has largely focused its efforts on the identification of recurrent karyotypic alterations, also known as clonal chromosomal aberrations (CCAs). The rationale of doing so seems simple: recurrent genetic changes are relevant for diseases or specific physiological conditions, while non clonal chromosome aberrations (NCCAs) are insignificant genetic background or noise. However, in reality, the vast majority of chromosomal alterations are NCCAs, and it is challenging to identify commonly shared CCAs in most solid tumors. Furthermore, the karyotype, rather than genes, represents the system inheritance, or blueprint, and each NCCA represents an altered genome system. These realizations underscore the importance of the re-evaluation of NCCAs in cytogenetic analyses. In this concept article, we briefly review the definition of NCCAs, some historical misconceptions about them, and why NCCAs are not insignificant “noise,” but rather a highly significant feature of the cellular population for providing genome heterogeneity and complexity, representing one important form of fuzzy inheritance. The frequencies of NCCAs also represent an index to measure both internally- and environmentally-induced genome instability. Additionally, the NCCA/CCA cycle is associated with macro- and micro-cellular evolution. Lastly, elevated NCCAs are observed in many disease/illness conditions. Considering all of these factors, we call for the immediate action of studying and reporting NCCAs. Specifically, effort is needed to characterize and compare different types of NCCAs, to define their baseline in various tissues, to develop methods to access mitotic cells, to re-examine/interpret the NCCAs data, and to develop an NCCA database.

## Background

Large scale –omics have revealed the surprising observation that stochastic alterations at various genetic and non-genetic levels are overwhelming [1]. These levels range from gene mutation, copy number variation, transcription regulation, protein degradation, molecular pathway switching/genetic network rewiring, and karyotype changes, to disease progression and therapeutic response [2]. Such a high “noise” level challenges the rationale and strategy of searching solely for the recurrent molecular patterns in the name of understanding bio-specificity-defined mechanisms. This approach has unconsciously altered the key feature of the Biosystems we are studying [1]. Interestingly, the seemingly random “non clonal chromosome aberrations,” or NCCAs, have long been observed in both normal and disease conditions, and the importance of studying this stochasticity or “noise” at the karyotype level has been vigorously pushed by a few groups (Table 1). However, the overall response to this effort has been rather limited due to the current cytogenetic practice, in which the main effort is the documentation of recurrent or “clonal chromosome aberrations,” or CCAs. In this perspective, we will briefly review NCCAs, an important but often ignored topic in molecular cytogenetics. We will first compare the concept of NCCAs and CCAs, challenge the general notion of solely focusing on recurrent patterns that ignore the majority of cases with NCCAs, and introduce new types of NCCAs; we then will discuss the importance of using NCCAs to measure system instability. As the karyotype represents a new type of genetic information, the system inheritance, NCCAs are not “noise;” rather, they function as the basis of genome heterogeneity, which is the essential form of genomic complexity and one of the pre-conditions for many diseases. We will further apply the cellular evolutionary mechanism to illustrate the disease process, and integrate the elevated NCCAs and NCCA/CCA cycle as the key condition for cellular adaptation, as well as the price to pay for the trade-off. Finally, since the variable karyotype serves as a good model to study fuzzy inheritance, the cytogenetics and cytogenomics field has positioned itself to tackle the important issue of how genome alteration unifies other types of molecular analyses, especially with the power of monitoring large number of individual cells within a defined cellular population. We thus call for the immediate action of documenting and reporting the data on NCCAs and their dynamic patterns in normal and disease conditions. This effort will have a profound impact beyond the field of cytogenetics and cytogenomics, as these stochastic alterations are also important for gene mutation and epigene regulation studies.

**Table 1:** Examples of NCCAs related studies

		Ref. #
(I) General description and classification of NCCAs	Wolman SR et al. (1984). *Can Genet Cytogenet* 16: 49-64	[22]
	Casalone R et al. (1992). *Hum Genet* 90(1–2): 71-8	[86]
	Mandahl N et al. (1994). *Genes Chromosomes Cancer* 9(3): 207-15	[87]
	Atkin NB et al. (2003). *Cytogenet Cell Genet* 101(2): 99-102	[25]
	Roschke AV et al. (2003). *Cancer Res* 63(24): 8634-47	[45]
	Heng HH et al. (2006). *J Cell Biochem* 98: 1424-35	[17]
	Heng HH et al. (2006). *Genome* 49: 195-204	[18]
	Mitelman F (2006). *NCI, NIH, USA*	[42]
	Bayani J et al. (2007). *Semin Cancer Biol* 17(1): 5-18	[15]
(II) Variable forms of NCCAs have been reported		
Various numerical/ structural aberrations	Erenpreisa J et al. (2005). *Cell Biol Int* 29(12): 1005-11	[88]
	Erenpreisa J et al. (2010). *Oncogene* 29(40): 5447-51	[89]
Chromosome fragmentations (C-Frag)	Stevens JB et al. (2007). *Cancer Res* 67 (16): 7686-94	[90]
Sticky chromosomes	Heng HH et al. (2013). *Cytogenet Genome Res* 139(3): 144-57	[66]
Chromosome bridge	Gisselsson D (2001). *Atlas Genet Cytogenet Oncol Haematol* 5(3): 236-43	[91]
Defective mitotic figures (DMF)	Heng H et al. (1988). *Mutat Res* 199(1): 199-205	[32]
	Smith L et al. (2001). *Proc Natl Acad Sci U S A* 98(23): 13300-5	[92]
Genome/ karyotype/ chromosome chaos	Heng HH (2006). *J Cell Physiol* 208: 461-72	[16]
	Duesberg P (2007). *Sci Am* 296(5): 52-9	[39]
	Heng HH (2007). *FASEB: Nuclear Structure and Cancer*	[55]
	Liu G et al. (2014). *Cell Cycle* 13(4): 528-37	[62]
Karyoplast budding	Walen KH (2005). *Cell Biol Int* 29(12): 1057-65	[93]
Giant nuclei	Walen KH (2010). *Cell Biol Int* 34(8): 867-72	[94]
	Heng HH et al. (2013). *Cytogenet Genome Res* 139(3): 144-57	[66]
	Liu G et al. (2014). *Cell Cycle* 13(4): 528-37	[62]
	Zhang S et al. (2014). *Oncogene* 33(1): 116-28	[69]
(III) Mechanism of NCCAs	Heng HH et al. (2006). *J Cell Physiol* 208: 461-72	[16]
	Heng HH et al. (2011). *Genomics* 98(4): 242-52	[49]
	Vincent MD (2011). *Adv Cancer Res* 112: 283-350	[95]
	Stepanenko AA et al. (2012). *Biopolymers and Cell* 28(4):267-80	[96]
	Huang S (2013). *Cancer Metastasis Rev* 32(3–4): 423-48	[97]
	Duesberg P & McCormack (2013). *Cell Cycle* 12(5):783-802	[98]
	Horne SD et al. (2014). *Front Genet* 134(9): 2074-87	[65]
	Horne SD et al. (2015). *eLS:* 1-9	[12]
(IV) Significance of NCCAs; they are linked to:		
Chromosomal instability (CIN)	Barrios L et al. (1991). *Hum Genet* 88: 39-41	[28]
	Gisselsson D et al. (2001). *Proc Natl Acad Sci U S A* 98(22): 12683–8.	[99]
	Ye C et al. (2007). *Cytogenet Genome Res* 18: 237-46	[11]
	Foster N et al. (2009). *Cytogenet Genome Res* 127(1): 9-20	[38]
	Ye C et al. (2009). *J Cell Physiol* 219: 288-300	[59]
	Heng HH et al. (2013). *Cytogenet Genome Res* 139(3): 144-57	[66]
	Jackson TR et al. (2013). *Cell Cycle* 12(3): 430-41	[70]
Gene defects	Shen KC et al. (2005). *Cancer Res* 65: 8747-53	[9]
	Heng HH et al. (2006). *J Cell Phyisol* 208: 461-72	[16]
	Sharpless NE et al. (2001). *Mol Cell* 8(6): 1187-96	[46]
Environmental stress	Stevens JB et al. (2011). *Cell Death Dis* 2: e178 DOI: 10.1038/cddis.2011.60.	[100]
Disease conditions/ prediction, as well as normal tissue/aging processes	Hsu TC (1983). *Hereditas* 98: 1-9	[33]
	Biesterfeld S et al. (1994). *J Clin Pathol* 47(1): 38-42	[101]
	Spitz MR et al. (1994). *Cancer Detect Prev* 18: 299-303	[102]
	Hagmar L et al. (1998). *Recent Results Cancer Res* 154: 177-84	[31]
	Bonassi S et al. (2000). *Cancer Res* 60: 1619-25	[29]
	Karashima T et al. (2000). *Cancer Genet Cytogenet* 120(2): 148-54	[103]
	López de Mesa R et al. (2000). *Cancer Genet Cytogenet* 121(1): 78–85.	[104]
	Kasahara K et al. (2002). *Cancer Genet Cytogenet* 137(1): 59-63	[105]
	El-Zein R et al. (2005). *Cancer Epidemiol Biomarkers Prev* 14: 748-52	[30]
	Kolusayin Ozar MO et al. (2005). *J Exp Clin Cancer Res* 24: 217-22	[34]
	Petersen I et al. (2009). *Lung Cancer* 65(3): 312-8	[106]
	Fenech M (2011). *Mutagenesis* 26(1): 63-7	[107]
	Heng HH et al. (2016). *Curr Genomics* (submitted)	[108]
Chemotherapy/radiation treatment (and they occur in the development of resistance)	Scott D et al. (1999). *Int J Radiat Biol* 75: 1	[36]
	Duesberg P et al. (2007). *Drug Resist Updat* 10(1–2): 51-8	[40]
	Heng HH et al. (2010). *Curr Drug Targets* 11: 1304-16	[56]
Evolutionary potential (both in vitro and in vivo)	Rancati G et al. (2008). *Cell* 135(5): 879-93	[109]
	Heng HH et al. (2009). *Bioessays* 31(5): 512-25	[48]
	Pearse AM et al. (2012). *Cancer Genet* 205(3): 101-12	[110]
	Potopova TA et al. (2013). *Cancer Metastasis Rev* 32(3–4): 377-89	[111]
	Stepanenko A et al. (2015). *Mutat Res* 771: 56-69	[58]

### NCCA or CCA? That is the question

The cytogenetic classification of karyotype aberrations has traditionally played a key role in genetic analysis and its applications in medical genetics. The establishment of normal karyotypes and the identification of major recurrent chromosomal aberrations have contributed to our understanding of the mechanism of many genetic diseases. This, in turn, has framed our approach in developing new prognostic and diagnostic methods. Additionally, studying patterns of karyotypic evolution has been useful in improving our grasp of organismal evolution [3–11]. To date, the majority of this achievement has been based on the analysis of CCAs; only in some specific cases, such as radiation-induced chromosomal breakages and chromosomal changes from patients with chromosomal instability syndromes, have the NCCAs involved been analyzed [12]. As for somatic polyploidy-diploid studies, many stochastic transitions generate NCCAs, even though they are often considered as tetraploid or near-diploid (Table 1). This can also apply to the B chromosome, as well as small supernumerary marker chromosomes [13, 14]. Prior to discussing this further, let us first briefly review the definition and classification of NCCAs and CCAs.

**Fig. 1:**
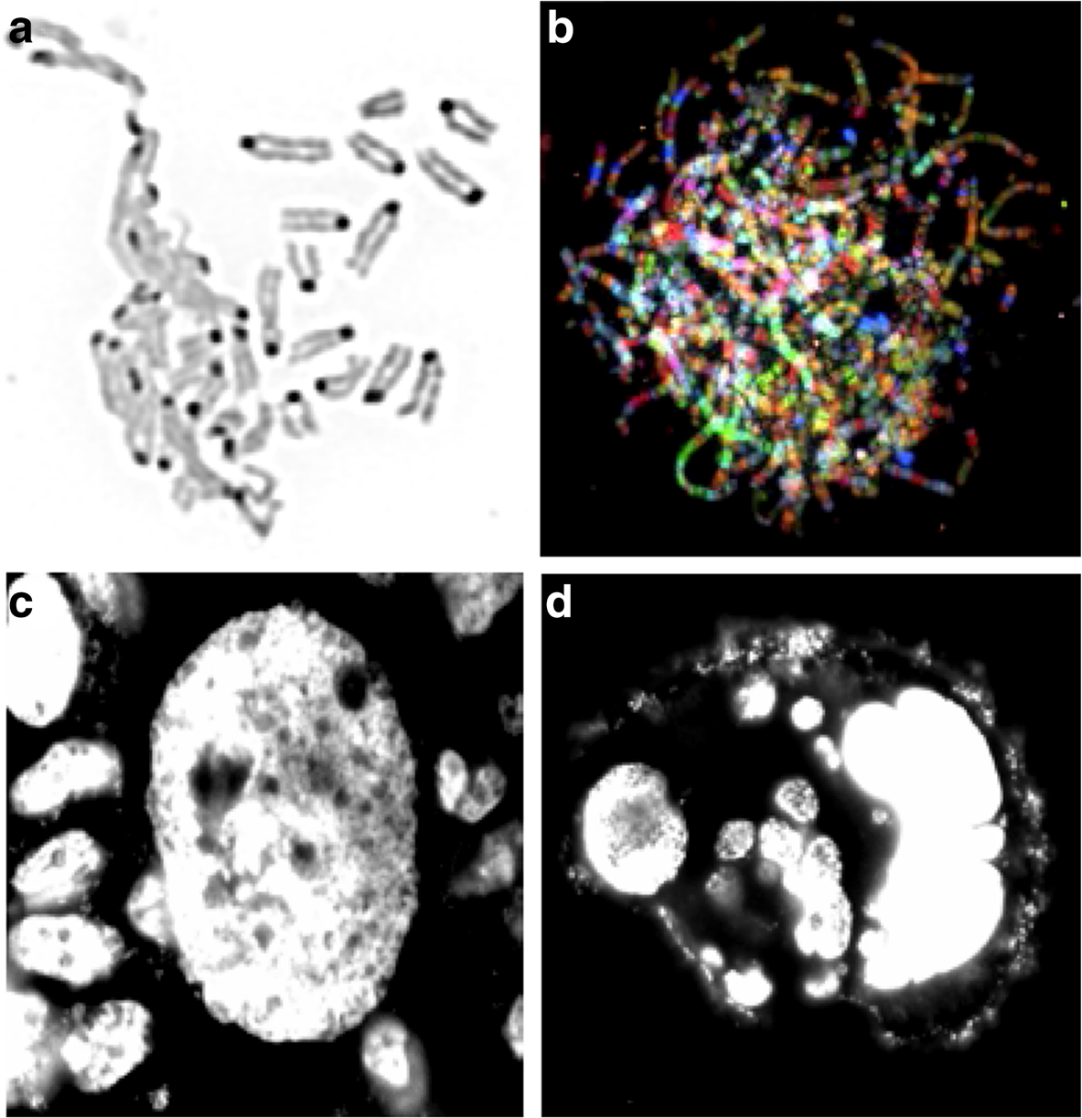
Examples of NCCAs: **a** DMF image (reversed DAPI image) detected from mouse cell culture. Left portion shows the de-condensed chromosomes are tangling together, while some normal condensed chromosomes are nearby. **b** SKY image of a chaotic genome detected from a Dox treated mouse cell. Each normal chromosome should have one unique color. However, for these massively re-organized chromosomes, there are multiple colors detected from each single chromosome, indicating the multiple events of chromosomal shattering and stitching. Note that there are many extremely long chromosomes. **c** An image of a giant nucleus (DAPI image) detected from HT-29 cells cultured in situ. Typical normal-sized nuclei are surrounding the giant nucleus. **d** An image of a cluster of cells derived from one giant nucleus. Since many of these cells are stochastically generated and display different amounts of DNA, these cells represent NCCAs when they enter into metaphase. Live imaging shows that there are continuous division /fusion events for unstable cancer cells, suggesting a new means of generating fuzzy inheritance
  In addition to being classified by their structural and numerical differences, CCAs can be further classified into different types. In the “watching karyotype evolution in action” experiments, there are many short-lived transitional CCAs (i.e., those CCAs detectable before the establishment of a cell line), and late-stage, more stable CCAs (which serve as the featured aberrations for the cell line or the specific cancer sample). In the clinic, there are some signature CCAs which can be used as a common marker for a given disease, such as the Philadelphia chromosome for chronic myelogenous leukemia or CML, and an extra chromosome 21 for Down’s syndrome. In general, CCAs dominate in the stepwise microcellular phase of cancer evolution. More information on NCCAs/CCAs can be found in our previous publications [11, 16–18].

- Definitions and classifications:
  Current cytogenetics defines a clonal chromosome aberration (CCA) as a given chromosome aberration which can be detected at least twice within 20 to 40 randomly examined mitotic figures. Based on this definition, the frequency of CCA needs to be higher than 5-10 % in an examined cell population. In literature, however, when a CCA is reported, researchers often refer to aberrations with frequencies that are over 30 %. Using the cut-off line of CCAs, a non-clonal chromosome aberration (NCCA) should refer to aberrations observed at a frequency of less than 5 %. According to our experience, we usually examine 50–100 mitotic figures when scoring NCCAs and CCAs, and therefore, 4 % is used as the cut-off (i.e., less than 2 in 50 mitotic cells examined); this is done even though, theoretically, the cut-off line could be 1 % or lower (i.e., if more than 100 mitotic figures are used).
  NCCAs can be classified into structural and numerical types [11, 12, 15]. There are increased structural types of NCCAs being reported (See Table 1, and Fig 1). Within the punctuated macro-cellular evolutionary phase, massive amounts of NCCAs can be detected, often coupled with complex chromosomal aberrations.

b. NCCAs have been considered as insignificant genetic “noise”:
  Starting from late 1960s, most cytogenetic methods (i.e. various chromosomal banding, FISH, SKY/m-FISH, and CGH) are designed to identify specific chromosomal abnormalities (both in individual chromosomes and particular regions of a given chromosome). These major technological achievements have pinpointed many genetic aberrations of specific diseases, including the linking of the Philadelphia chromosome (Ph) to CML [19], as well as that of recurrent translocations to an array of subtypes of blood cancer [20]. When molecular cloning became dominant in the field of human genetics, karyotyping became a powerful tool for the process of gene cloning. It is no surprise that more attention has been focused on the recurrent abnormalities, as they should host key genes important for diseases. In contrast, the large amount of NCCAs detected from the majority of cases have been considered as insignificant “noise” [21]. Most cytogenetic reports do not include NCCA data. The art of karyotype studies seems to be in the identification of the recurrent patterns from the background of NCCA noise. For example, in some earlier cytogenetic reports, despite the fact that NCCAs were commonly observed, and that they were clearly more common in tumor-derived tissue than in normal culture, it was concluded that a considerable fraction of breast cancers are composed predominantly of diploid cells [22]. These reports have been considered by many as evidence to support the viewpoint that many cancers do not display abnormal karyotypes. In addition to the limited identification power of G-banding and the bias of methods that authors had mentioned, the ignorance of NCCAs was a main reason for this. Newer references have stated that, for the majority of solid tumors, karyotype aberration is a common feature [23–26]. Cancer genome sequencing projects have also confirmed that chromosome level abnormality is overwhelming in solid cancers [27], and that far more structural alterations than gene mutation/copy number variations are involved when comparing primary tumors to metastatic cancer.

c. NCCAs have been sporadically but persistently studied by some investigators:
  Nevertheless, limited research on NCCAs persists, due to the fact that they are overwhelmingly present (Table 1). This is especially true when their frequencies are very high, and when the karyotypes involve highly complex translocations. For example, elevated NCCAs have been observed from blood cultures of cancer patients, particularly when subsequent to radiation or chemotherapy. It is also well known that for cancers featuring chromosomal instability, this increased chromosomal instability is reflected by elevated NCCAs [8, 9, 26–38]. Of course, most of the aneuploidy in solid tumors is NCCA type [25, 39–41], and most of the chromosomal aberrations detected during chemotherapy regimens are nonclonal and unbalanced (>75 %). Interestingly, for all those examined tumor cases reported so far, the majority of them display different aberrations, which should ultimately lead to the realization that NCCAs are fundamentally important and common CCAs are only detectable in a minority of cases [42, 43]! In fact, in current databases, NCCAs are under-represented due to publication bias. Most researchers will only write a paper when the recurrent chromosomal aberrations are identified, leaving most data of NCCAs unreported.
  The reason that the research community ignores NCCAs is simple: the complex cases of NCCAs do not fit with the prediction of the gene mutation theory of genetic diseases. In the case of cancer, following the successful cloning of the bcr-abl gene from CML, many researchers became and still are convinced that they must find the recurrent pattern of most solid tumors, not knowing the distinctive evolutionary dynamics between CML and the majority of cancers [44]. In addition, based on the gene mutation theory of cancer (wherein for each cancer type, there should be only a few key driver gene mutations), the large scale of stochastic genome alterations does not make any sense [1, 44]. Equally misleadingly, a CGH profile based on a mixed cell population does indeed wash off many NCCAs. As soon as some common recurrent CCAs are identified (even at lower frequencies), researchers no longer consider the large number of co-existing NCCAs, as if considering the NCCAs as “noise” can bring closure to a specific investigation. Furthermore, people have reasoned (or hoped) that if we have more precise identification methods in the future, such as multiple color SKY and high-resolution CGH, as well as more high quality samples, then we should be able to find the limited recurrent pattern. To date, current cytogenetic/cytogenomic technologies have failed to identify the magic pattern, and researchers continue to hope for more powerful future methods. Based on the overwhelming amount of NCCAs and the diversity of CCAs detected, why not ask a simple question: what if there are no simple and common CCAs for the majority of cancers?
  Clearly, there is a paradox regarding NCCAs and CCAs in the literature. On one hand, elevated NCCAs can be detected in both tumor samples and an individual’s circulating lymphocytes; in the latter case, this is often coupled with an increased cancer risk following exposure to irradiation and carcinogens (see Table 1). In fact, clinical samples display highly dynamic karyotypic data [42, 43], and even for such well-established cell lines as those of the NCI-60 drug-screening panel, NCCAs are common [45]. Additionally, increased molecular studies have linked specific gene mutations to stochastic chromosomal aberrations [9, 16, 46, 47]. On the other hand, the majority of researchers are not interested in this highly significant avenue, despite the fact that they have failed to identify key recurrent patterns for a vast majority of cases. We realized that systematic research about the NCCA and its dynamic relationship with CCAs within an evolutionary context needed to be used to change the popular yet incorrect attitude towards system heterogeneity. Only when we have the correct framework to appreciate NCCAs will people take action. Prior to our series of studies, these seemly random chromosomal changes failed to link the common mechanisms. Since our NCCA research has been ongoing for over a decade, only some highlights will be mentioned here. More information can be found from our recent publication [1].
  Following the trace of NCCA/CCA dynamics through the use of various experimental systems to watch somatic cell evolution in action, and especially upon the detection of NCCAs from normal individuals and elevated NCCAs from various disease conditions, we realized the importance of these types of aberrations. Even though the frequency of stochastic translocations is low for many cases, there are many different types of NCCAs, and some of them have been ignored for decades (Table 1) (Fig 1). Collectively, the frequencies of NCCAs are high. It was thus clear to us that we needed to promote new research based on NCCA/CCA cycles and the evolutionary dynamics between potential and end products, rather than solely focusing on highly limited CCAs. To achieve this goal, we need demonstrate the biological significance of stochastic genome alterations in the context of genetic information and genome instability-mediated somatic cell evolution.

### NCCAs are not insignificant “noise” but rather a highly significant feature of the genome system

- Genome (not gene) defines genetic blueprint:
  According to the genome theory, genetic information can be divided into different main types: the gene-defined “parts inheritance” (i.e., how gene codes for specific protein) and the genome-defined “system inheritance” or blueprint (i.e., how genome codes the genetic network for a given species) [1]. Since the relationship of all gene interaction is defined by the physical matrix among genes (the interactive potential under various environmental conditions), the order of the genes along an individual chromosome and among different chromosomes determines the genetic networks within 3-dimensional nuclei [1, 48, 49]. Interestingly, the maintenance of karyotype-mediated genetic information is through sexual reproduction [50–52].
  The gene and genome relationship mimics the relationship between parts and the whole. Not only there is no simple accumulative relationship between gene and genome; quantitatively speaking, genome level change often is much greater than individual gene mutations, as the genome functions as a package unit for evolutionary selection [1, 48]. All altered genomes, like NCCAs, represent altered systems.

b. NCCAs represent genome level heterogeneity and can serve as an index for genome instability:
  Knowing that the essential function of the karyotype is to encode key genetic information (genetic blueprint), it is easier to appreciate the importance of karyotype variations, as these represent the altered system inheritance. Our research has demonstrated that NCCAs are not “noise” after all, as NCCAs-formed genome heterogeneity is a key feature of the biological system that also functions as a layer of complexity [1, 53, 54]. Using cancer evolution as an example, almost all genetic and non-genetic factors that can contribute to carcinogenesis can ultimately (either directly or indirectly) be linked to elevated NCCAs . During the punctuated phase of macro-cellular evolution, NCCAs are dominant, coupled with a large number of transitional CCAs. Only during the stepwise phase of micro-cellular evolution are CCAs persistent. Importantly, different runs of evolution often lead to different CCAs, as many CCAs can serve as the end product of evolution. In other words, most cancers will display different karyotypes or combinations of NCCAs and variable CCAs. Since heterogeneity is the essential condition for the evolutionary process itself, researchers who seriously study cancer should not consider NCCAs as noise. Many conclusions from linear model systems that significantly reduced the system heterogeneity are not suitable to clinical reality, which underscores the importance of including NCCA-mediated heterogeneity in the study of human diseases.
  One important realization is that the frequencies of NCCAs can be used as a reliable index to measure the genome instability of a given cell population or cell line. Both internal instability and drug-induced instability can be measured. The NCCAs/CCAs cycles have been observed from all key transition stages of the cancer evolution, from immortalization and transformation, to metastasis and drug resistance [38, 55–59]. Increased attention should be paid to more common and complex diseases or illness conditions.

c. The function of genome heterogeneity: evolutionary potential:
  First, elevated levels of NCCAs were linked to tumorigenicity, which again agree with the common observation that multiple factors that contribute to cancer can be linked to elevated NCCAs [59]. Then, the punctuated phase of cancer evolution was linked to the transcriptome dynamics [60]. Furthermore, outliers were linked to evolutionary dominance under stress, which ultimately demonstrated the importance of NCCAs in cancer evolution [61]. In fact, when genome chaos was induced by chemotherapy drugs, the massive NCCAs became apparent as the key for cancer cells to survive and quickly evolve to form much more stable and simpler genomes [62]. Putting all of these experiments/observations together, NCCAs represent the evolutionary potential by creating new genome systems with altered transcriptomes and phenotypes.

d. Mechanisms of maintaining NCCAs-mediated heterogeneity and complexity:
  In addition to stress-induced NCCA frequencies (of both genetic and environmental origins), there are internal mechanisms which maintain a certain degree of heterogeneity. First, heterogeneity is not just a bad thing for a biosystem caused by stress, but an important adaptation mechanism (even though too much is certainly not good). We realized that a certain degree of NCCAs is essential for normal cellular function under stress. This idea explains the observation that there are moderate or even high levels of NCCAs in many healthy tissues, especially when adaptation is needed (such as the aging process, wound healing, tissue regeneration, and inflammation). Second, to search for the mechanism of the internal basis for generating and maintaining NCCAs in the first place, we searched for a new type of inheritance at the somatic cell level, called fuzzy inheritance. It turns out that genetic information is not as precise as we have believed, but rather fuzzy. More specifically, for most somatic cell traits, it is the range of genetic change (such as the degree of NCCAs), rather than a specific change (like a specific karyotype), that can be inherited, especially for less stable cellular populations such as some cancer cell populations. Such a genetic mechanism likely serves as the basis for the genetic heterogeneity of cancer. When coupled with somatic cell evolution, precise prediction becomes much less so. Interestingly, fuzzy inheritance combined with somatic cell evolution nicely explains the issue of missing heritability [1, 54, 63–65]).
  When faced with environmental dynamics, fixed genetic information would have a great disadvantage. The better strategy would be to inherit an evolutionary potential which contains an array of possible plans, rather than a fixed specific plan. The separation of germline and somatic cells allow somatic cells to display the highest level of change by increasing the heterogeneity (for adaptation), while the precise mechanism maintains the system inheritance through the germline cells among generations of individuals. Even though the developmental process and the aging process can bring about a great deal of somatic cellular-level changes through the passing of fuzzy inheritance, the species will not be impacted, as most of these genome level changes will be washed away during sexual reproduction. Somatic cell adaptation and germline constraint forms a beautiful balance for short-term adaptation and long-term species’ existence. It is clear that heterogeneity, functioning as a new layer of complexity, plays an important role for achieving such balance.
  It is interesting to point out that under the high levels of stress, the chaotic genome (including that of some giant cells with hundreds of chromosomes) can push fuzzy inheritance to the maximum [1, 12, 54, 65, 66]. In such a situation, the generated cells can display a highly altered genome, and even display stem cell-like phenotypes, possibly achieving this by drastically switching from mitotic to meiotic machineries [66–70]. This drastic change is caused by diverse survival mechanisms of the genome. According to the genome theory, when stress is too high to survive, re-organization of the genome becomes the method of choice. In this case, precise inheritance is less useful. In contrast, highly fuzzy inheritance can produce a large number of potential survivors, most of which are distinctively different, and a tiny portion of them will save the day. That is what macro-cellular evolution is all about.

### What action is needed for the cytogenetics community to study and report NCCAs?

Over 60 years have passed since TC Hua developed the hypotonic method to analyze the human karyotype [71]. Despite the fact that recurrent karyotype aberrations have been used in medical genetics and played important role in genetic diagnosis, cytogenetics has been thought of by some as not as important as molecular genetics, as the karyotype only represents the carrier of the genes rather than gene itself. In fact, there are some premature suggestions of using molecular methods such as array CGH and DNA sequencing to replace karyotype analysis. Now, realizing that karyotype functions as the master that determines the structure of the genetic network, and that NCCAs play an important role in genome heterogeneity/complexity, which is absolutely not replaceable by DNA-level profiling, it is obvious that studying the mechanism of how the karyotype and its heterogeneity works both in somatic cell and organismal evolution should be much important than illustrating the functions of individual genes. Interestingly, the success of cytogenetics in the past has greatly enhanced molecular genetics (chromosomal aberrations have helped in the identification of many cancer genes, for example). The domination of molecular genetics has unfortunately reduced the significance of cytogenetics. Now, the success and limitation of gene-centric research will finally put the future cytogenetics back into the driver’s seat, as the genome-defined system is much more important than the sum of all parts of genes, and it is genome replacement-mediated macro-cellular evolution (rather than gene mutation-led micro-cellular evolution) that represents the general mechanism for diseases like cancer. While exciting, it is very challenging to develop a new technological platform to advance the field based on the importance of the karyotype and NCCAs. Followings are some suggestions:

- Systematic characterization of different types of NCCAs
  Currently, when discussing NCCAs, most researchers refer to non-recurrent chromosomal translocations and aneuploidy. However, as we listed in Table 1, there are many more types of chromosomal abnormalities belonging to this category, including defective mitotic figures (DMF), sticky chromosomes, chromosome fragmentations (C-Frag), and highly diverse chaotic genomes (including unstable giant nuclei, in which a series of transitions occurs, cycling from polyploidy to altered diploid chromosomes through multipolar and bipolar mitoses). Our recent publications have summarized these accumulated data over decades [12, 66]. The list of types of NCCAs can grow further when more investigators are interested in NCCAs. In addition to chromosome-based abnormalities, there are many strange-looking interphase nuclei that clearly are not normal judged by their irregular morphology. Interestingly, there are many novel types of chromosomal aberrations following the induction of genome chaos. Many of them seem not very stable; therefore, it could be hard to observe in the more stable systems. Further characterization is clearly needed.

b. Compare and integrate different types of NCCAs
  Due to the large number and diverse types of NCCAs, it is necessary to compare their contribution to evolutionary potential as well as disease phenotypes. For example, when measuring chromosomal instability (CIN), we initially focused on structural NCCAs when the system was highly unstable. The limitation of such analyses is that the contribution of aneuploidy has been ignored. Improved analyses need to include all types of NCCAs, but how do we score them in the overall contribution? Should we treat them equally or not? Knowing that structural change might have more profound impact than lower levels of aneuploidy [59], a combinational score system is needed. Such complication is far beyond aneuploidy. The contribution of one single chromatid break is likely to contribute less than a translocation, but is this true? How many breaks should be equal to one translocation when measuring their genetic contribution towards CIN? How about the abnormality distribution within cell the population? Is an individual cell with multiple abnormalities more harmful than many cells displaying only chromosomal aberration? And what about the relationship between DMF and translocation, between C-Frag and aneuploidy, and between simple translocation and complex translocations?
  There are some further complications. The tolerance of chromosomal abnormalities or CIN seems to differ among different tissue types. Liver tissue displays a high level of tolerance to polyploidy and aneuploidy, and embryogenic cells seem to be able to tolerate more chaotic genomes. Should we modify the score criteria when studying different types of tissue? This issue also applies to different cancer models, wherein a certain degree of CIN will make a huge difference in terms of tumorigenesis. Now, knowing that the two phases of cancer evolution are involved, should we use a different calculation matrix when scoring chromosomal aberration in different phases of cancer evolution? Finally, it is known that most of the specific NCCAs will be eliminated (for those cells that can precisely pass their inheritance by inheriting the same karyotype, the CCAs will be emergent); as such, how do we predict the survival rate of each type of NCCA? For example, many cells displaying a lower degree of aneuploidy will have greater chance to survive than those with a highly re-shuffled genome with multiple translocations. But when they survive, these cells with complex genomes will have higher impact on cancer evolution.

c. Establish the baseline of NCCAs for various tissues
  One important technical issue is to differentiate real noise (e.g. the cell culture and preparation artefacts like over-spread metaphases) from NCCAs. In addition to standardizing protocols, attention is needed to reduce the technical variability. Interphase FISH could be used to study the baseline of aneuploidy, for example. Studies are also needed to quantify the contribution from the cell culture process to the baseline of various types of NCCAs. Similarly, various tissues, as well as those of differently-aged of individuals, should be used to establish the baseline. A comparison between in vivo samples (using sensitive DNA-cytometry methods) and in vitro culture (using classical cytogenetic methods) is also needed.

d. Develop methods to access mitotic cells
  One of the biggest limitations for karyotype analysis is the requirement of mitotic figures. The need of dividing cells excludes the usage of fixed biomaterials such as fixed pathological slides. In addition, in the case of many fresh tumor samples, karyotype analysis often requires a short-term cell culture (i.e., a few days, and especially if NCCAs will be scored, as more mitotic figures are necessary), which might introduce further chromosomal changes. To solve this problem, methods are needed to promote the presence of mitotic cells. Alternatively, interphase FISH can be used with a panel of probes to monitor aneuploidy and to infer structural changes. To indirectly monitor genome chaos, it is also possible to use a few whole chromosome paints to study the organized chromosome using interphase FISH.

e. Re-examine/interpret the NCCAs data
  To illustrate NCCAs’ relevance in the clinic, association studies are needed to link the types and frequencies of NCCAs to some clinical features. We have illustrated the linkage between overall genome instability (reflected by the level of NCCAs) and the formation of secondary cancer [72]. Similarly, we have linked elevated NCCAs in circulating lymphocytes to various cancer types and their dynamics during treatments, as well as to some other illness conditions such as Gulf War Illness [[54, 66] unpublished data]. This supports previous observations that elevated frequencies of NCCAs in an individual’s blood can be linked to increased cancer risk following exposure to irradiation [73]. In fact, Dr. TC Hsu has hypothesized that genetic instability in the human population contributes to many cancer and other diseases, and that when challenged, the cells of persons with mildly defective repair systems may show a higher rate of chromosome aberrations than those of persons with stable repair systems [33]. With the realization that increased CIN is not just due to defective repair systems, but also the active response to stress as the mechanism of cellular adaptation[1, 54, 65], many more molecular mechanisms will be linked to CIN-mediated diseases as the result of somatic cell evolution and its trade-off. NCCAs-defined CIN should become the central subject for future research to illustrate disease mechanisms, and more importantly, NCCA/CCA profiling should be used to design diagnosis and treatment methods and for monitoring clinical outcomes. One important example is the use of the NCCAs to follow disease progression in cancer patients. Our experimental data has shown that drug resistance is highly associated with elevated NCCAs. In fact, even in the case of the successful story of using imatinib to treat CML patients, when the disease enters into the blast crisis stage, designated by high frequencies of NCCAs, imatinib is no longer useful [44]. Clearly, profiling patients’ NCCA/CCA pattern is important for designing the treatment options. Another important issue is to link the NCCA/CCA contribution to the status of the individual’s somatic mosaicism [74–76], as the degree of somatic mosaicism is closely linked to the phenotypes [77].

f. Develop an NCCA database
  For the majority of cytogenetic reports, the data of NCCAs are largely missing, despite the fact that many well-known structures such as ring chromosomes and chromosomal bridges are actually NCCAs. It is important to report NCCAs and establish a database. Such a database will serve multiple purposes. First, it will expand the list of types of NCCAs. Second, it will record the frequencies of different types of NCCAs and all types of NCCAs for normal individuals, for specific disease types, and for various tissue types. . Third, it will encourage the re-examination of published reports to collect the data, and initiate efforts to examine previous available samples. One interesting implication is to test the possibility that some balanced translocations might have no phenotype in parents, but could contribute to instability in offspring (i.e., the offspring could have the same balanced translocation as well as increased NCCAs). Furthermore, some CCAs (like balanced translocations) in conjunction with additional NCCAs could display an improved or worsened phenotype. Fourth, it will promote the integration of these data with other molecular databases, such as integration with databases of DNA sequence, copy number variation, small supernumerary marker chromosomes, and various cytogenetic databases [43, 78–80].

## Conclusion

In closing, genome system instability is the ultimate link between many diseases and their genetic and environmental contributing factors. The genome serves as the evolutionary platform that links gene/epigene interaction and multiple levels of omics [1]. Using the types and frequencies of NCCAs, and the dynamic relationship between NCCA and CCA, evolutionary potential can be monitored either genetically or environmentally [81], as all stress responses can be reflected by the level of system instability. This evolutionary mechanism of diseases can unify diverse molecular mechanisms, and reconcile the difficulty of clinical prediction based only on the genetic profile. Of equal importance, the significance of NCCAs will emphasize the ultimate importance of studying heterogeneity in biology, including heteromorphisms and euchromatic variants [54, 82–85]. Welcome to the age of genome- (karyotype-) based cytogenetic/genomic research!
